# Ultrasensitive Biosensor with Hyperbolic Metamaterials Composed of Silver and Zinc Oxide

**DOI:** 10.3390/nano11092220

**Published:** 2021-08-28

**Authors:** Shuhan Chen, Shiqi Hu, Yichen Wu, Dingnan Deng, Yunhan Luo, Zhe Chen

**Affiliations:** 1 School of Physics and Electronic Engineering, Jiaying University, Meizhou 514015, China; w756430347@163.com (Y.W.); deenan@jyu.edu.cn (D.D.); 2 Key Laboratory of Optoelectronic Information and Sensing Technologies of Guangdong Higher Education Institutes, College of Science and Engineering, Jinan University, Guangzhou 510632, China; hushiqi47@163.com (S.H.); thzhechen@jnu.edu.cn (Z.C.)

**Keywords:** surface plasmon resonance, hyperbolic metamaterials, silver and zinc oxide, sensitivity, figure of merit

## Abstract

We propose a hyperbolic metamaterial-based surface plasmon resonance (HMM-SPR) sensor by composing a few pairs of alternating silver (Ag) and zinc oxide (ZnO) layers. Aiming to achieve the best design for the sensor, the dependence of the sensitivity on the incidence angle, the thickness of the alternating layer and the metal filling fraction are explored comprehensively. We find that the proposed HMM-SPR sensor achieves an average sensitivity of 34,800 nm per refractive index unit (RIU) and a figure of merit (FOM) of 470.7 RIU^−1^ in the refractive index ranging from 1.33 to 1.34. Both the sensitivity (S) and the FOM show great enhancement when compared to the conventional silver-based SPR sensor (Ag-SPR). The underlying physical reason for the higher performance is analyzed by numerical simulation using the finite element method. The higher sensitivity could be attributed to the enhanced electric field amplitude and the increased penetration depth, which respectively increase the interaction strength and the sensing volume. The proposed HMM-SPR sensor with greatly improved sensitivity and an improved figure of merit is expected to find application in biochemical sensing due to the higher resolution.

## 1. Introduction

Surface Plasma Resonance (SPR) sensors have become a promising method in the field of food safety, drug screening and biological sensors over the last two decades [1,2,3]. To excite SPR, two configurations have usually been employed, namely Kretschmann configuration and grating coupling configuration [4]. In the Kreschmann configuration, spectral scan and angular scan interrogation are usually employed to measure the reflected signal [5]. Although the angular scan interrogation is possible to achieve higher signal-to-noise, the spectral scan has the advantages of low cost, easy fabrication and a compact fiber sensor [6]. 

Currently, various materials are added to the structure of traditional prism-coupled SPR biosensors to achieve ultra-high detection sensitivity, ultra-high detection accuracy and low detection threshold. For example, metallic nanoparticles and complex nanostructures have been used to enhance the sensitivity of SPR sensors [7,8]. Nanomaterials, such as TiO_2_ and graphene, have been deposited to modify the metal for exciting long-range surface plasmon [9,10].

Recently, hyperbolic metamaterials (HMM), which have been demonstrated to enhance the performance of SPR sensors distinctly, are one of the most concerned metamaterials with the real part of the permittivity components having opposite signs [11,12,13]. For instance, the gold/Al_2_O_3_ multilayered structure was fabricated as HMM-based sensors to achieve a sensitivity of 30,000 nm/RIU and an FOM of 509 RIU^−1^ [14]. An Ag/TiO_2_ HMM-based fiber plasmonic sensor was fabricated to achieve a sensitivity of 9000 nm/RIU and an FOM of 230.8 RIU^−1^, which has the advantage of miniaturization and integration [15]. Among a lot of semiconductors, ZnO as a wide band gap semiconductor was found to have high thermal and chemical stability, and thus it is an ideal material to protect the metallic layer from oxidation [16].

In this study, an HMM-SPR sensor composed of alternating Ag/ZnO layers is investigated. The ZnO layer is used to protect the Ag layer from oxidation. The HMM structure composed of Ag/ZnO multi-layers is demonstrated to greatly improve the sensitivity of the SPR sensor. The effects of the incidence angle, alternating layer thickness and metal filling factor on the sensitivity of the sensor are studied using the transfer matrix method (TMM) [17]. The underlying physical reason for the higher performance is analyzed by using finite element method [18]. The improvement is attributed to the enhanced electric field amplitude and the increased penetration depth, which respectively increase the interaction strength and the sensing volume. With optimized parameters, the average S of 34,800 nm/RIU and FOM of 470.7 RIU^−1^ are achieved in the range of 1.33 to 1.34 RIU.

## 2. Model and Theory

**Figure 1:**
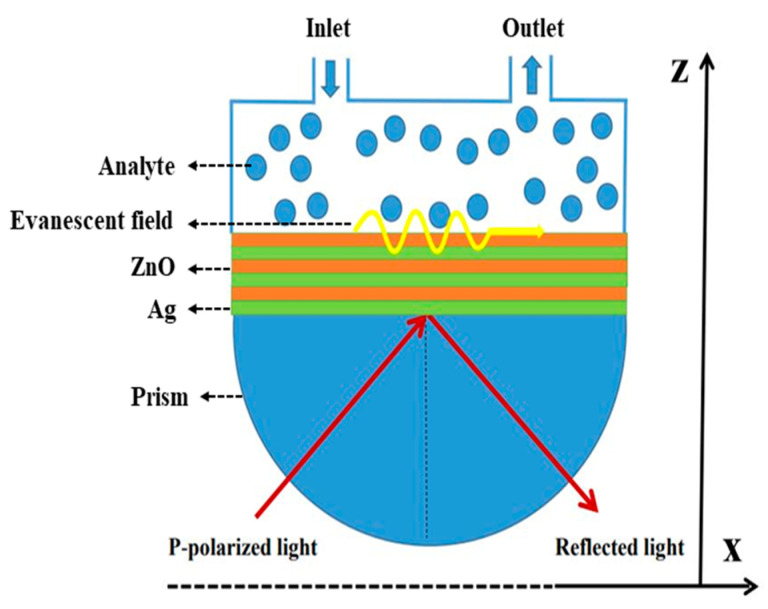
Schematic diagram of the proposed SPR sensor with hyperbolic metamaterial composed of Ag/ZnO.

In the proposed structure, the SPR sensor based on HMM composed of Ag/ZnO is shown in Figure 1. The Kretschmann configuration is used to excite the surface plasmon wave, where a BK7 prism is used as the coupling element. The Ag/ZnO bilayer in the structure has a thickness approximate 30 nm, which is much smaller than the exciting wavelength (500–2000 nm). Therefore, the alternating Ag/ZnO layers can be assumed to be a uniform medium.

The effective medium theory (EMT) is used to evaluate the parallel and perpendicular component of equivalent dielectric tensors of the HMM [19] with the following formulas:

(1) $$\varepsilon_{x}=\frac{d_{\mathrm{Ag}}\varepsilon_{\mathrm{Ag}}+d_{\mathrm{ZnO}}\varepsilon_{\mathrm{ZnO}}}{d_{\mathrm{Ag}}+d_{\mathrm{ZnO}}}$$

(2) $$\varepsilon_{z}=\frac{d_{\mathrm{Ag}}+d_{\mathrm{ZnO}}}{d_{\mathrm{Ag}}/\varepsilon_{\mathrm{Ag}}+d_{\mathrm{ZnO}}/\varepsilon_{\mathrm{ZnO}}}$$

where $d_{\mathrm{Ag}}$ and $d_{\mathrm{ZnO}}$ denote the thickness, while $\varepsilon_{\mathrm{Ag}}$ and $\varepsilon_{\mathrm{ZnO}}$ denote the dielectric permittivity of the Ag and ZnO layer, respectively. The $\varepsilon_{\mathrm{Ag}}$ is obtained according to Ref. [20]. The $\varepsilon_{\mathrm{ZnO}}$ is obtained according to Ref. [21], using the following formula:

(3) $$n^{2}=2.81418+\frac{0.87968\lambda^{2}}{\lambda^{2}-0.3042^{2}}-0.00711\lambda^{2}$$

where λ denotes the wavelength. The refractive index of the sensing medium is set as $n_{s}=1.33.$ The refractive index of BK7 prism ($n_{p}$) is obtained according to the formula as follows:

(4) $$n_{p}{}^{2}-1=\frac{1.03961212\lambda^{2}}{\lambda^{2}-0.00600069867^{2}}+\frac{0.231792344\lambda^{2}}{\lambda^{2}-0.0200179144^{2}}+\frac{1.01046945\lambda^{2}}{\lambda^{2}-103.560653^{2}}$$

To solve the proposed multilayer systems, the transfer matrix method is employed due to its simplicity and flexibility. For the *N*-layer model in this study, the propagation characteristic is described by transfer matrix *S*_m_ [22], i.e.,

(5) $$S=\left[\begin{array}{cc} S_{11} & S_{12}\\ S_{21} & S_{22} \end{array}\right]=\left(\prod_{m=1}^{N}I_{\left(m-1\right)m}L_{m}\right)\cdot I_{N\left(N+1\right)}$$

where the interface matrix for each interface in the structure is defined by

(6) $$I_{mn}=\frac{1}{t_{mn}}\left[\begin{array}{cc} 1 & r_{mn}\\ r_{mn} & 1 \end{array}\right]$$

where $t_{mn}$ and $r_{mn}$ are the reflection and transmission coefficients at the interface *mn,* which can be obtained by the Fresnel formula. For the TM-polarized mode, we can get the amplitude of reflection coefficient $r_{m}$ and transmission coefficient $t_{m}$ at the *m*th layer:

(7) $$t_{m}=\frac{2n_{m-1}\cos\theta_{m-1}}{n_{m-1}\cos\theta_{m}+n_{m}\cos\theta_{m-1}}$$

(8) $$r_{m}=\frac{n_{m-1}\cos\theta_{m}-n_{m}\cos\theta_{m-1}}{n_{m-1}\cos\theta_{m}+m\cos\theta_{m-1}}$$

where *θ* is the incident angle. The layer matrix through layer *m* is described by

(9) $$L_{m}=\left[\begin{array}{cc} e^{-i\emptyset_{m}} & 0\\ 0 & e^{i\emptyset_{m}} \end{array}\right]$$

where $\emptyset_{m}$ is the layer phase thickness as the wave traverses the layer *m*.

## 3. Results and Discussion

**Figure 2:**
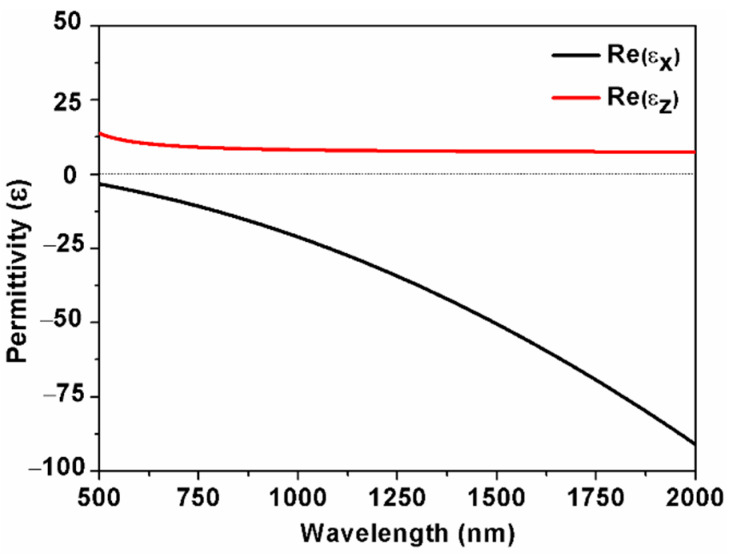
The effective permittivity of Ag/ZnO HMM calculated by EMT.

The effective permittivity calculated with EMT is plotted in Figure 2. The real part of the permittivity with *x* and *z* components show opposite signs for λ > 500 nm, which indicates that the alternating Ag/ZnO multilayered structure displays hyperbolic dispersion in this region. The modes in HMM cannot be directly excited due to the momentum mismatch. Therefore, a BK7 prism is used to couple the spatial light into the HMM structure and realize the match of the two wave-vectors [13].

The TMM is used to calculate the reflection spectra of the HMM-SPR and traditional Ag-SPR, and their S, full width at half maximum (FWHM), and the FOM and depth of resonance dip (DRD) are further calculated [23]. For the Ag-SPR sensor, an Ag film with a thickness of 50 nm is used to replace the Ag/ZnO multi-layers in the structure, shown as Figure 1. For the HMM-SPR sensor, the pair thickness of Ag/ZnO is *d* = 30 nm, and the metal filling fraction is *f* = 0.5. The pair number is set to be 3 based on our early simulation for optimization. 

**Figure 3:**
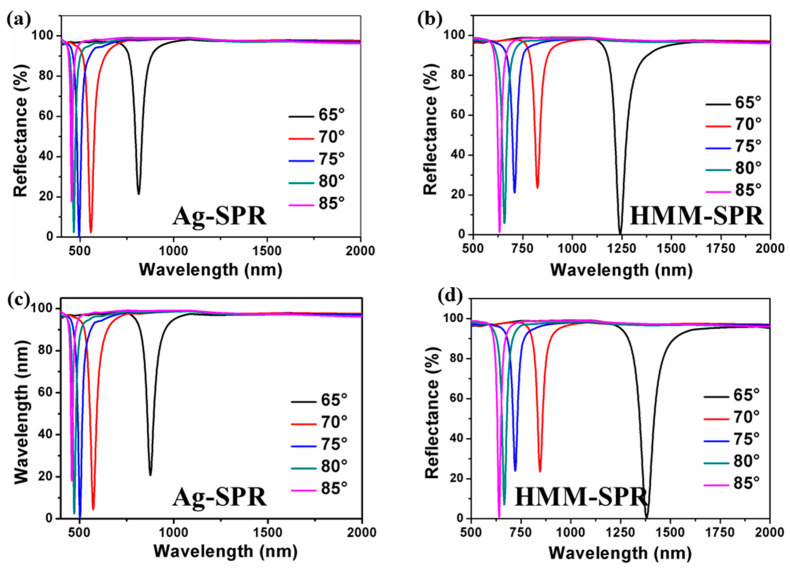
The dependence of reflectance for Ag-SPR and HMM-SPR for SRI = 1.330 (**a**,**b**), 1.335 (**c**,**d**) with different incident angles.

With the surrounding refractive index (SRI) increasing from 1.330 to 1.335, the reflection spectra for the Ag-SPR sensor under different incidence angles are shown in Figure 3a,c, while the reflection spectra for the HMM-SPR sensor at different incidence angles are shown in Figure 3b,d, respectively. When the SRI equals 1.33, the resonant wavelengths are 633, 658, 709, 824, and 1242 nm corresponding to the incidence angles of 85°, 80°, 75°, 70°, and 65°. It can be observed that the resonant wavelength is red-shifted as the incidence angle is increased. 

In order to further investigate the mechanism of performance improvement, the finite element method (FEM) is used to calculate the optical field intensity with the distance of the device for both sensors under the incidence angle of 65°. In the calculations, the top and bottom of the computational domain are set as periodic boundary conditions. The input power of port in the left side is set as 1 W/m. Adaptive meshing with a maximum element size of 30 nm has been used. 

**Figure 4:**
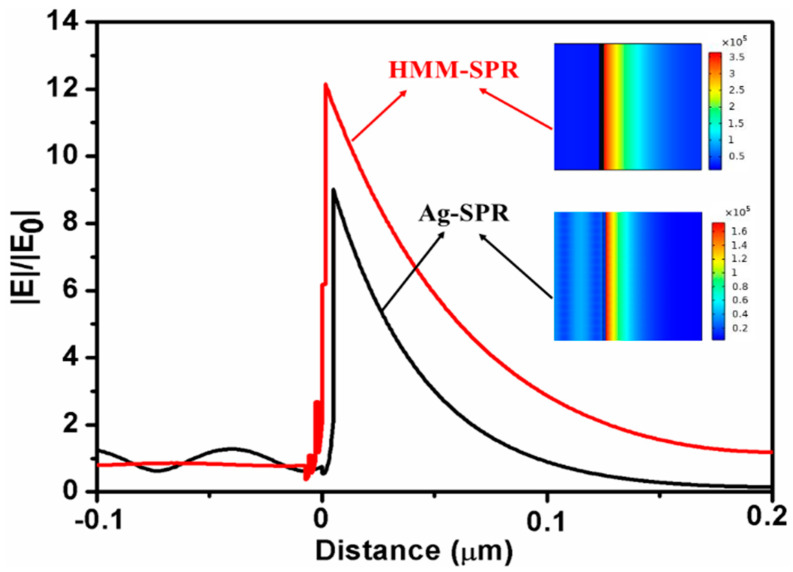
The distribution of normalized optical electric field |E|/|E_0_| along with the device distance.

Figure 4 shows the distributions of the normalized optical electric field |E|/|E_0_| along with the device distance for HMM-SPR and Ag-SPR sensors. It can be seen that the electric field amplitude and the increased penetration depth for the HMM-SPR sensor are both improved distinctly compared with Ag-SPR sensors. The higher sensitivity could be attributed to the enhanced electric field amplitude and the increased penetration depth, which respectively increase the interaction strength and the sensing volume.

**Figure 5:**
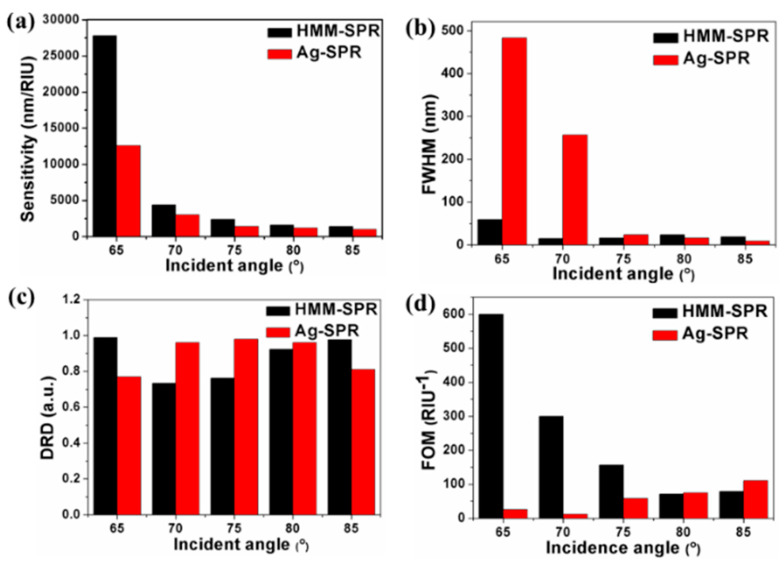
(**a**) S, (**b**) FWHM, (**c**) DRD and (**d**) FOM distributions with the incidence angle for the HMM-SPR and Ag-SPR sensors, respectively.

To quantitatively analyze the sensing performance, the S, FWHM, DRD and FOM are plotted in Figure 5. The *S* increases when the incidence angle decreases. At an incidence angle of 65° with the SRI region ranging from 1.330 to 1.335, the average S of the HMM-SPR and Ag-SPR sensors is 27,800 and 12,600 nm/RIU, and the average FOM is 600 and 26 RIU^−1^, respectively. The HMM-SPR sensor shows a sensitivity enhancement of 120.6% over the Ag-SPR sensor.

**Figure 6:**
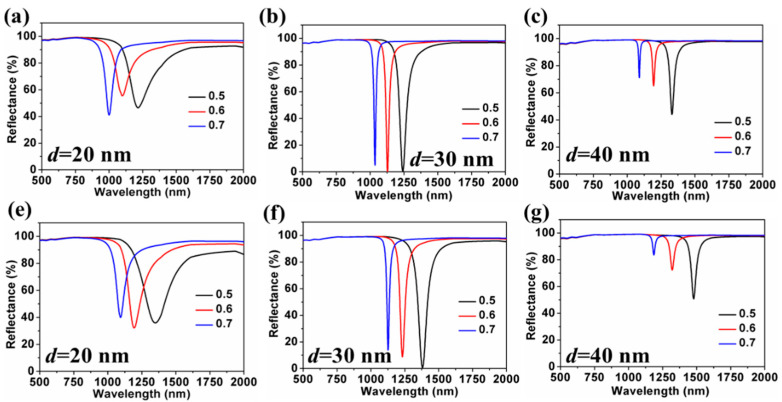
The dependence of the reflectance on *d* and *f* for HMM-SPR with SRI = 1.330 (**a**–**c**) and 1.335 (**e**–**g**), respectively.

**Figure 7:**
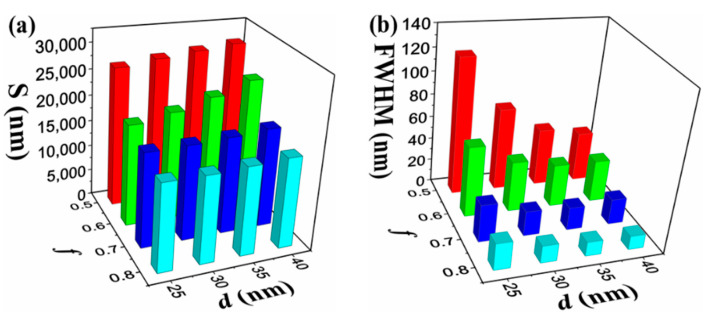
The dependence of *S* (**a**) and FWHM (**b**) on the *f* and *d* in the case of SRI changing from 1.330 to 1.335.

Figure 6a,c and Figure 6e,g show the dependence of reflection spectrums on thickness of the alternating layer (*d*) and the metal filling fraction (*f*) for HMM-SPR with SRI = 1.330 and 1.335 respectively. The resonance wavelengths of the sensors are all shown to be blue-shifted with an increase in *f*. The influence of *d* and *f* on the S and FWHM of HMM-SPR are shown in Figure 7. The S increases with decreasing *f* and increases with increasing *d*. However, the FWHM is shown to increase with decreasing *f* and decrease with increasing *d*. By comparing the reflection spectrum in Figure 6 and S distribution in Figure 7, it can be seen that the higher S for HMM-SPR can be attributed to the higher effective refractive index for SPR at a longer resonant wavelength.

**Figure 8:**
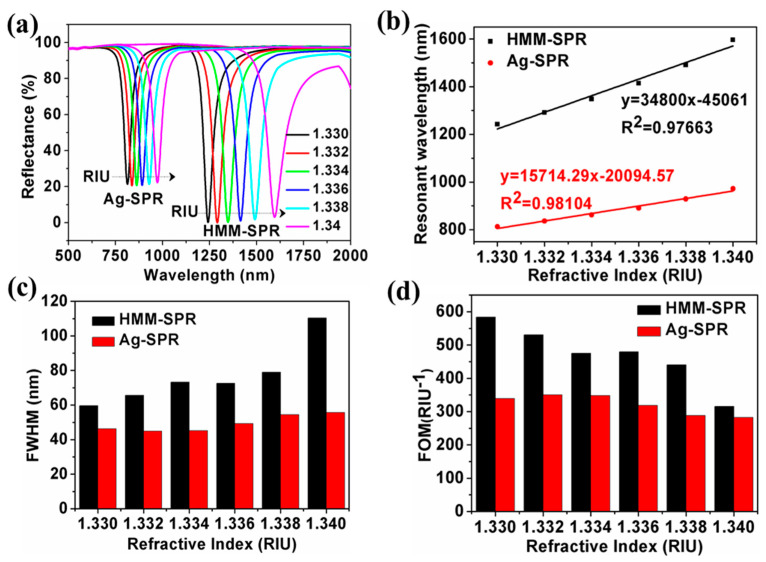
The dependence of the reflectance on the refractive index for the HMM-SPR (**a**) and Ag-SPR sensor (**b**). Comparison of the resonant wavelength (**c**). Comparison of S (**d**).

Figure 8a shows the reflection spectra of HMM-SPR (*d* = 30 nm, *f* = 0.5) and Ag-SPR at different SRI, respectively. The depth of the reflection dip for the HMM-SPR sensor is significantly larger than that for the Ag-SPR sensor. The resonant wavelength is redshifted with the increasing SRI. Figure 8b shows the dependence of the resonant wavelength on RIU for both sensors. The resonant wavelength is shown to be linear with the RIU. The slopes of the fitting lines represent the average S of the sensors. With the SRI ranging from 1.33 to 1.34, the average S of the HMM-SPR and Ag-SPR sensors is 34,800 nm/RIU and 15,714 nm/RIU, respectively. A sensitivity enhancement up to 121.4% was achieved. The FWHM and FOM are shown in Figure 8c,d. The average FOM are 470.7 RIU^−1^ and 321.3 RIU^−1^ for HMM-SPR and Ag-SPR, respectively.

Table 1 summarizes the results of various SPR sensors concerning the S and FOM from the previous reports, including the HMM-Au grating, HMM-SPR-Prism Nanorod HMM-Prism, HMM-SP-FMF and Au/Ag multilayer-prism SPR sensors. It is shown that the Ag/ZnO HMM-SPR sensor proposed in this paper possesses a much higher S and FOM.

## 4. Conclusions

An ultrasensitive HMM based SPR biosensor composed of alternating Ag/ZnO layers is numerically investigated using TMM. The underlying physical reason for the higher performance is analyzed using FEM. The higher sensitivity for HMM-Sensor is attributed to the enhanced electric field amplitude and the increased penetration depth, which respectively increased the interaction strength and the sensing volume. The effects of the incidence angle, alternating layer thickness and metal filling factor on the performance of the sensor are studied. The S and FOM are demonstrated to increase with a decrease in the incidence angle. The S is demonstrated to increase with decreasing *f* at a certain *d* and to increase with increasing *d* at a certain *f*. With the SRI region ranging from 1.33 to 1.34, an average S of 34,800 nm/RIU and FOM of 470.7 RIU^−1^ for the proposed HMM-SPR sensor are achieved. The proposed biosensor with greatly improved S and FOM is expected to find application in ultrasensitive biochemical sensing field.

## Figures and Tables

**Table 1:** Comparison of various SPR sensors.

Configuration	Detection Range (RIU)	S (nm/RIU)	FOM (RIU^−1^)	Reference
Ag/ZnO HMM	1.33–1.34	34,800	470.7	This paper
HMM-Au Grating	1.3333–1.3336	30,000	590	[14]
Nanorod HMM-Prism	-	30,000	330	[24]
HMM Fiber	1.33–1.40	9000	230.8	[15]
Au/Ag multilayer-Prism	~1.3558	4154	56.9	[25]
Ag/ZnO bilayers	1.30–1.37	3161	-	[16]

## Data Availability

The data presented in this study are available on request from the corresponding author upon reasonable request.
